# Supplementary material for: The Effect of Cushioned Centrifugation, with and without Enzymatic Reduction of Viscosity, on the Motility Pattern and Kinematic Parameters of Dromedary Camel Bull Spermatozoa
Source: Animals (Basel). 2023 Aug 22;13(17):2685. doi: 10.3390/ani13172685 (PMC10487258; doi:10.3390/ani13172685)
Supplement: Supplementary file 1 [file animals-13-02685-s001.zip › Monaco et al_2023_Suppl. Table S2.pdf]

Supplementary Table S2. Effects of centrifugation (900 g x 20 min at RT) with and without cushion fluid on kinematic parameters of dromedary camel bull spermatozoa.

VCL: curvilinear velocity; **VAP**: average path velocity; **VSL**: straight line velocity; **STR**: straightness; **LIN**: linearity; **WOB:** wobble **ALH**: amplitude of lateral head displacement; **BCF:** beat cross frequency.

VCL (µm/s)	Pre_Centr Mean ± S.E.	Centr_Con Mean ± S.E.	Centr_Cush Mean ± S.E.	Pre_Centr vs Centr_Con	Pre_Centr vs Centr_Cush	Centr_Con vs Centr_Cush
Motile	76.43 ± 10.56	92.58 ± 11.45	110.6 ± 12.56	0,0859	**<0.0001**	**0.0013**
Progressive	83.07 ± 10.77	106.42 ± 10.31	119.24 ± 11.63	0.6576	**0.0007**	0.5382
Rapid progressive	78.77 ± 12.88	106.37 ± 14.37	117.97 ± 9.76	0.9981	0.1696	1
Medium Progressive	83.92 ± 10.44	106.42 ± 10.51	118.96 ± 11.72	0.7135	**0.002**	0.6367
Non Progressive	21.33 ± 1.11	21.19 ± 1.63	21.16 ± 1.27	1	1	1

VAP (µm/s)	Pre_Centr Mean ± S.E.	Centr_Con Mean ± S.E.	Centr_Cush Mean ± S.E.	Pre_Centr vs Centr_Con	Pre_Centr vs Centr_Cush	Centr_Con vs Centr_Cush
Motile	32.74 ± 4.44	38.04 ± 4.17	44.76 ± 4.66	0.9376	**<.0001**	**0.0263**
Progressive	37.38 ± 3.97	43.37 ± 3.73	48.86 ± 4.34	0.9103	**0.0045**	0.3189
Rapid progressive	40.84 ± 3.62	43.10 ± 3.09	50.05 ± 3.18	1	0.4853	0.0874
Medium Progressive	37.23 ± 3.96	43.22 ± 3.82	48.73 ± 4.37	0.9133	**0.0041**	0.325
Non Progressive	9.79 ± 0.64	9.87 ± 1.23	8.46 ± 0.80	1	0.5109	0.8884

VSL (µm/s)	Pre_Centr Mean ± S.E.	Centr_Con Mean ± S.E.	Centr_Cush Mean ± S.E.	Pre_Centr vs Centr_Con	Pre_Centr vs Centr_Cush	Centr_Con vs Centr_Cush
Motile	15.73 ± 2.15	18.73 ± 1.87	21.94 ± 1.80	0.9233	**<.0001**	0.2596
Progressive	17.68 ± 2.19	21.42 ± 1.70	24.09 ± 1.65	0.8531	**<.0001**	0.5931
Rapid progressive	31.69 ± 3.91	36.59 ± 2.63	42.13 ± 2.79	0.9996	0.2638	0.0581
Medium Progressive	17.16 ± 2.03	20.87 ± 1.69	23.54 ± 1.60	0.8157	**<.0001**	0.5438
Non Progressive	4.18 ± 0.56	4.49 ± 0.84	3.72 ± 0.59	1	0.9985	0.9424

STR (%)	Pre_Centr Mean ± S.E.	Centr_Con Mean ± S.E.	Centr_Cush Mean ± S.E.	Pre_Centr vs Centr_Con	Pre_Centr vs Centr_Cush	Centr_Con vs Centr_Cush
Motile	46.88 ± 1.32	49.93 ± 1.22	49.95 ± 1.88	0.4289	0.7746	1
Progressive	47.29 ± 1.56	50.38 ± 1.23	50.78 ± 2.01	0.595	0.6658	1
Rapid progressive	78.53 ± 4.73	85.32 ± 0.76	84.12 ± 0.55	0.9741	0.9955	0.7914
Medium Progressive	46.17 ± 1.20	49.21 ± 1.09	49.82 ± 1.81	0.4909	0.5535	1
Non Progressive	40.61 ± 2.07	44.59 ± 3.03	43.95 ± 1.69	0.8786	0.9414	1

LIN (%)	Pre_Centr Mean ± S.E.	Centr_Con Mean ± S.E.	Centr_Cush Mean ± S.E.	Pre_Centr vs Centr_Con	Pre_Centr vs Centr_Cush	Centr_Con vs Centr_Cush
Motile	21.36 ± 0.70	21.96 ± 0.81	21.24 ± 1.04	1	1	0.9998
Progressive	21.59 ± 0.57	21.84 ± 0.61	21.70 ± 1.06	1	1	1
Rapid progressive	37.83 ± 2.86	38.22 ± 2.40	38.05 ± 1.82	1	1	1
Medium Progressive	20.99 ± 0.45	21.24 ± 0.58	21.26 ± 0.99	1	1	1
Non Progressive	18.16 ± 1.70	20.34 ± 2.42	16.79 ± 1.65	0.9097	1	0.5928

WOB (%)	Pre_Centr Mean ± S.E.	Centr_Con Mean ± S.E.	Centr_Cush Mean ± S.E.	Pre_Centr vs Centr_Con	Pre_Centr vs Centr_Cush	Centr_Con vs Centr_Cush
Motile	45.12 ± 0.79	43.85 ± 1.10	42.61 ± 0.79	0.9996	0.7754	0.8817
Progressive	45.67 ± 0.62	43.22 ± 0.94	42.78 ± 0.77	0.8119	0.4008	1
Rapid progressive	47.69 ± 2.25	44.61 ± 2.70	45.12 ± 2.01	0.5507	0.9981	1
Medium Progressive	45.36 ± 0.65	43.10 ± 0.95	42.75 ± 0.77	0.8714	0.5996	1
Non Progressive	44.48 ± 1.74	45.70 ± 2.09	39.29 ± 1.78	1	0.8691	0.0883

ALH (µm)	Pre_Centr Mean ± S.E.	Centr_Con Mean ± S.E.	Centr_Cush Mean ± S.E.	Pre_Centr vs Centr_Con	Pre_Centr vs Centr_Cush	Centr_Con vs Centr_Cush
Motile	2.07 ± 0.26	2.54 ± 0.29	2.93 ± 0.32	0.7809	**0.001**	**0.0305**
Progressive	2.31 ± 0.24	2.87 ± 0.27	3.16 ± 0.30	0.6013	**0.0054**	0.4305
Rapid progressive	2.20 ± 0.22	2.69 ± 0.32	2.98 ± 0.21	0.9979	0.0527	0.9998
Medium Progressive	2.31 ± 0.24	2.87 ± 0.27	3.16 ± 0.30	0.6221	**0.0076**	0.5391
Non Progressive	0.87 ± 0.03	0.84 ± 0.05	0.81 ± 0.04	0.9997	0.7375	0.9975

BCF (Hz)	Pre_Centr Mean ± S.E.	Centr_Con Mean ± S.E.	Centr_Cush Mean ± S.E.	Pre_Centr vs Centr_Con	Pre_Centr vs Centr_Cush	Centr_Con vs Centr_Cush
Motile	8.68 ± 0.65	9.19 ± 0.49	10.41 ± 0.45	0.9993	**<.0001**	**0.002**
Progressive	10.19 ± 0.41	10.69 ± 0.34	11.49 ± 0.29	0.9991	**<.0001**	0.3421
Rapid progressive	13.38 ± 1.24	11.14 ± 0.83	11.87 ± 0.65	0.997	0.9986	0.9992
Medium Progressive	10.14 ± 0.40	10.67 ± 0.34	11.46 ± 0.30	0.9971	**<.0001**	0.3045
Non Progressive	2.30 ± 0.27	2.24 ± 0.46	1.89 ± 0.40	1	0.4555	0.9989
