# Supplementary material for: The Effect of Cushioned Centrifugation, with and without Enzymatic Reduction of Viscosity, on the Motility Pattern and Kinematic Parameters of Dromedary Camel Bull Spermatozoa
Source: Animals (Basel). 2023 Aug 22;13(17):2685. doi: 10.3390/ani13172685 (PMC10487258; doi:10.3390/ani13172685)
Supplement: Supplementary file 1 [file animals-13-02685-s001.zip › Monaco et al_2023_Suppl. Table S3.pdf]

**Supplementary Table S3.** Effects of enzymatic reduction of seminal plasma viscosity with Papain 0.1 mg/mL (5 min) + E64 10µM before and after a cushioned centrifugation procedure (900 g x 20 min at RT) on kinematic parameters of dromedary dromedary camel spermatozoa. **VCL**: curvilinear velocity; **VAP**: average path velocity; **VSL**: straight line velocity; **STR**: straightness; **LIN**: linearity; **WOB:** wobble **ALH**: amplitude of lateral head displacement; **BCF:** beat cross frequency.

| | | Con | Pap | Centr_Con | Centr_Pap | P Values | | | | | |
|---|---|---|---|---|---|---|---|---|---|---|---|
| | | Mean ± S.E. | Mean ± S.E. | Mean ±S.E. | Mean ± S.E. | Con Vs Pap | Con Vs Centr_Con | Con Vs Centr_Pap | Pap Vs Centr_Con | Pap Vs Centr_Pap | Centr_Con Vs Centr_Pap |
| **VCL (µm/s)** | | | | | | | | | | | |
| | Motile | 66.82 ± 5.08 | 120.63 ± 12.23 | 86.08 ± 6.00 | 120.33 ± 10.16 | **<.0001** | **0.0043** | **<.0001** | **0.0148** | 1 | **0.0011** |
| | Progressive | 103.78 ± 3.98 | 142.51 ± 9.02 | 120.55 ± 4.32 | 141.30 ± 7.56 | **0.0002** | **0.001** | **<.0001** | **0.0374** | 1 | **0.0048** |
| | Rapid progr | 104.93 ± 6.49 | 138.89 ± 7.43 | 114.51 ± 8.53 | 138.78 ± 7.99 | 0.1921 | 1 | 0.1051 | 0.904 | 1 | 0.9514 |
| | Medium Progr | 103.05 ± 4.25 | 141.77 ± 9.44 | 114.62 ± 7.93 | 140.58 ± 7.64 | **0.0004** | 0.9847 | **<.0001** | 0.0626 | 1 | **0.0053** |
| | Non Progr | 43.82 ± 1.23 | 43.19 ± 1.08 | 43.25 ± 0.88 | 43.21 ± 1.00 | 1 | 1 | 1 | 1 | 1 | 1 |
| **VAP (µm/s)** | | | | | | | | | | | |
| | Motile | 31.10 ± 2.33 | 52.75 ± 5.35 | 39.40 ± 3.02 | 51.22 ± 4.04 | **<.0001** | **0.0064** | **<.0001** | **0.0089** | 1 | **0.0004** |
| | Progressive | 47.57 ± 2.15 | 61.85 ± 4.00 | 53.75 ± 2.67 | 60.13 ± 2.99 | **0.0006** | 0.0728 | **0.0017** | 0.1117 | 1 | 0.0898 |
| | Rapid progr | 49.43 ± 2.41 | 61.90 ± 3.18 | 54.90 ± 2.93 | 60.44 ± 2.60 | **0.0272** | 0.7049 | **0.0072** | 0.7407 | 1 | 0.7473 |
| | Medium Progr | 46.92 ± 2.23 | 61.49 ± 4.21 | 53.10 ± 2.71 | 59.77 ± 3.11 | **0.0022** | 0.0979 | **0.0041** | 0.1132 | 1 | 0.0694 |
| | Non Progr | 20.82 ± 1.12 | 18.74 ± 0.83 | 20.32 ± 1.01 | 18.34 ± 1.00 | 0.9802 | 1 | 0.9898 | 0.9946 | 1 | 0.9936 |
| **VSL (µm/s)** | | | | | | | | | | | |
| | Motile | 14.64 ± 1.37 | 22.98 ± 2.28 | 18.96 ± 1.67 | 24.10 ± 1.92 | **0.0001** | **<.0001** | **<.0001** | 0.107 | 0.9999 | **0.0062** |
| | Progressive | 23.80 ± 1.54 | 28.26 ± 1.80 | 26.37 ± 1.97 | 29.48 ± 1.72 | **0.0303** | 0.9682 | 0.0854 | 0.9988 | 0.9998 | 0.8115 |
| | Rapid progr | 39.20 ± 1.97 | 48.11 ± 2.51 | 42.55 ± 2.39 | 47.34 ± 2.42 | 0.1408 | 0.9503 | 0.1296 | 0.8687 | 1 | 0.8643 |
| | Medium Progr | 21.24 ± 1.28 | 25.88 ± 1.69 | 23.70 ± 1.43 | 26.88 ± 1.40 | 0.0845 | 0.8014 | **0.012** | 0.8808 | 0.9999 | 0.3197 |
| | Non Progr | 8.91 ± 0.68 | 6.96 ± 0.53 | 8.75 ± 0.61 | 7.11 ± 0.54 | 0.6888 | 1 | 0.9124 | 0.7229 | 1 | 0.846 |
| **STR (%)** | | | | | | | | | | | |
| | Motile | 45.53 ± 1.54 | 43.49 ± 1.67 | 46.26 ± 1.49 | 46.73 ± 1.94 | 0.9903 | 1 | 1 | 0.7104 | 0.3077 | 1 |
| | Progressive | 49.29 ± 2.05 | 47.21 ± 2.17 | 49.37 ± 2.21 | 50.23 ± 2.50 | 1 | 1 | 1 | 0.9999 | 0.7471 | 1 |
| | Rapid progr | 78.52 ± 0.85 | 77.61 ± 0.46 | 77.10 ± 0.79 | 77.88 ± 1.26 | 1 | 1 | 1 | 1 | 1 | 1 |
| | Medium Progr | 45.39 ± 1.87 | 43.76 ± 1.73 | 45.64 ± 1.59 | 46.23 ± 1.88 | 1 | 1 | 1 | 0.9998 | 0.7616 | 1 |
| | Non Progr | 42.88 ± 1.27 | 36.41 ± 1.80 | 41.97 ± 1.20 | 39.03 ± 1.43 | 0.2151 | 1 | 0.7873 | 0.3048 | 0.953 | 0.9248 |
| **LIN (%)** | | | | | | | | | | | |
| | Motile | 21.29 ± 0.94 | 19.22 ± 0.82 | 21.75 ± 0.83 | 20.36 ± 0.73 | 0.7859 | 1 | 1 | 0.3843 | 0.9859 | 0.9932 |
| | Progressive | 23.66 ± 1.27 | 20.91 ± 0.98 | 22.87 ± 1.35 | 21.77 ± 0.96 | 0.8767 | 1 | 0.9874 | 0.9977 | 0.9996 | 1 |
| | Rapid progr | 39.45 ± 2.49 | 35.46 ± 1.24 | 36.49 ± 1.92 | 35.10 ± 1.18 | 0.864 | 0.9983 | 0.9714 | 1 | 1 | 1 |
| | Medium Progr | 21.64 ± 1.17 | 19.28 ± 0.76 | 20.66 ± 0.84 | 20.01 ± 0.70 | 0.9595 | 1 | 0.9932 | 0.9922 | 0.9998 | 1 |
| | Non Progr | 19.96 ± 1.07 | 16.09 ± 0.97 | 19.60 ± 1.06 | 16.20 ± 0.98 | 0.357 | 1 | 0.6355 | 0.6616 | 1 | 0.6333 |

| | Con | Pap | Centr_Con | Centr_Pap | P Values | | | | | |
|---|---|---|---|---|---|---|---|---|---|---|
| | Mean ± S.E. | Mean ± S.E. | Mean ± S.E. | Mean ± S.E. | Con Vs Pap | Con Vs Centr_Con | Con Vs Centr_Pap | Pap Vs Centr_Con | Pap Vs Centr_Pap | Centr_Con Vs Centr_Pap |
| **WOB (%)** | | | | | | | | | | |
| Motile | 46.97 ± 1.34 | 44.17 ± 1.16 | 45.94 ± 1.04 | 43.86 ± 1.06 | 0.7644 | 0.9919 | 0.8208 | 0.8863 | 1 | 0.9375 |
| Progressive | 46.59 ± 1.49 | 44.05 ± 1.00 | 45.37 ± 1.30 | 43.55 ± 0.99 | 0.812 | 0.9997 | 0.4555 | 0.9998 | 1 | 0.9856 |
| Rapid progr | 49.92 ± 2.98 | 45.61 ± 1.59 | 47.10 ± 2.40 | 45.31 ± 1.57 | 0.924 | 0.9997 | 0.9724 | 1 | 1 | 1 |
| Medium Progr | 46.32 ± 1.41 | 44.00 ± 1.01 | 44.65 ± 1.15 | 43.49 ± 0.99 | 0.8656 | 0.9815 | 0.4562 | 1 | 1 | 0.9979 |
| Non Progr | 46.85 ± 1.70 | 43.68 ± 1.41 | 45.67 ± 1.66 | 41.66 ± 1.72 | 0.8998 | 1 | 0.8834 | 0.9999 | 0.9992 | 0.9701 |
| **ALH (µm)** | | | | | | | | | | |
| Motile | 1.92 ± 0.12 | 3.20 ± 0.29 | 2.39 ± 0.15 | 3.18 ± 0.25 | **0.0001** | **0.0137** | **<.0001** | **0.0247** | 1 | **0.0015** |
| Progressive | 2.81 ± 0.10 | 3.72 ± 0.21 | 3.22 ± 0.11 | 3.68 ± 0.19 | **0.0002** | **0.001** | **<.0001** | 0.0965 | 1 | **0.019** |
| Rapid progr | 2.73 ± 0.17 | 3.47 ± 0.15 | 3.07 ± 0.14 | 3.54 ± 0.20 | 0.2007 | 0.944 | 0.127 | 0.9373 | 1 | 0.9619 |
| Medium Progr | 2.80 ± 0.11 | 3.72 ± 0.22 | 3.23 ± 0.12 | 3.68 ± 0.19 | **0.0004** | **0.0018** | **0.0002** | 0.0762 | 1 | **0.0095** |
| Non Progr | 1.37 ± 0.03 | 1.37 ± 0.03 | 1.35 ± 0.02 | 1.35 ± 0.03 | 1 | 1 | 1 | 1 | 1 | 1 |
| **BCF (Hz)** | | | | | | | | | | |
| Motile | 8.94 ± 0.51 | 11.63 ± 0.75 | 9.78 ± 0.55 | 11.63 ± 0.48 | **<.0001** | 0.4974 | **<.0001** | **0.0031** | 1 | **<.0001** |
| Progressive | 12.04 ± 0.46 | 13.55 ± 0.56 | 12.18 ± 0.47 | 13.62 ± 0.39 | 0.3199 | 1 | **0.0077** | **0.0071** | 1 | **0.0001** |
| Rapid progr | 12.01 ± 0.72 | 14.15 ± 0.68 | 12.29 ± 0.86 | 13.74 ± 0.44 | 0.458 | 1 | 0.4027 | 0.6845 | 1 | 0.9357 |
| Medium Progr | 11.93 ± 0.43 | 13.35 ± 0.59 | 11.94 ± 0.44 | 13.34 ± 0.38 | 0.4014 | 1 | 0.1918 | 0.0608 | 1 | **0.0072** |
| Non Progr | 7.02 ± 0.40 | 5.95 ± 0.37 | 6.61 ± 0.30 | 5.74 ± 0.36 | 0.7797 | 0.9984 | 0.7474 | 0.9943 | 1 | 0.9553 |
