# Supplementary material for: The Effect of Cushioned Centrifugation, with and without Enzymatic Reduction of Viscosity, on the Motility Pattern and Kinematic Parameters of Dromedary Camel Bull Spermatozoa
Source: Animals (Basel). 2023 Aug 22;13(17):2685. doi: 10.3390/ani13172685 (PMC10487258; doi:10.3390/ani13172685)
Supplement: Supplementary file 1 [file animals-13-02685-s001.zip › Monaco et al_2023_Suppl. Table S1.pdf]

Supplementary Table S1. Effects of 35% Seminal Plasma incubation (45 min at 7°C) on the kinematic parameters of dromedary camel bull epididymal spermatozoa. **VCL**: curvilinear velocity; **VAP**: average path velocity; **VSL**: straight line velocity; **STR**: straightness; **LIN**: linearity; **WOB**: wobble **ALH**: amplitude of lateral head displacement; **BCF**: beat cross frequency.

**VCL (μm/s)**	**Control Mean ± S.E.**	**Control Time Mean ± S.E.**	**Seminal Plasma Mean ± S.E.**	**Con VS Con TIME**	**Con VS SP**	**Con TIME VS SP**
**Motile**	122.47 ± 12.16	115.83 ± 11.90	82.13 ± 8.24	n.s.	**0.0028**	**0.0008**
**Progressive**	133.53 ± 10.89	128.33 ± 9.26	109.63 ± 6.35	n.s.	n.s.	**0.0209**
**Rapid progressive**	128.28 ± 8.94	111.44 ± 16.18	93.57 ± 13.63	n.s.	**0.019**	**<0.0001**
**Medium Progressive**	134.21 ± 10.99	128.78 ± 9.44	97.42 ± 13.08	n.s.	n.s.	n.s.
**Non Progressive**	46.04 ± 1.27	46.18 ± 1.52	45.17 ± 1.44	n.s.	n.s.	n.s.

**VAP (μm/s)**	**Control Mean ± S.E.**	**Control Time Mean ± S.E.**	**Seminal Plasma Mean ± S.E.**	**Con VS Con TIME**	**Con VS SP**	**Con TIME VS SP**
**Motile**	54.16 ± 5.41	49.31 ± 4.71	35.56 ± 3.34	n.s.	**0.0003**	**0.0016**
**Progressive**	59.39 ± 4.85	54.66 ± 3.64	47.92 ± 2.42	n.s.	n.s.	n.s.
**Rapid progressive**	62.32 ± 5.11	48.79 ± 7.06	42.28 ± 5.77	n.s.	**0.0202**	n.s.
**Medium Progressive**	58.95 ± 5.07	54.72 ± 3.64	47.96 ± 2.44	n.s.	n.s.	n.s.
**Non Progressive**	18.69 ± 1.03	19.28 ± 0.94	19.70 ± 1.00	n.s.	n.s.	n.s.

**VSL (μm/s)**	**Control Mean ± S.E.**	**Control Time Mean ± S.E.**	**Seminal Plasma Mean ± S.E.**	**Con VS Con TIME**	**Con VS SP**	**Con TIME VS SP**
**Motile**	23.03 ± 2.10	22.03 ± 2.56	15.22 ± 1.71	n.s.	**0.0044**	**0.0005**
**Progressive**	25.83 ± 2.09	24.09 ± 2.60	21.36 ± 1.77	n.s.	n.s.	n.s.
**Rapid progressive**	47.94 ± 4.18	37.49 ± 5.42	31.95 ± 4.37	n.s.	**0.0203**	n.s.
**Medium Progressive**	23.56 ± 1.94	22.89 ± 2.31	20.35 ± 1.58	n.s.	n.s.	n.s.
**Non Progressive**	6.44 ± 0.45	6.62 ± 0.50	7.21 ± 0.63	n.s.	n.s.	n.s.

**STR (%)**	**Control Mean ± S.E.**	**Control Time Mean ± S.E.**	**Seminal Plasma Mean ± S.E.**	**Con VS Con TIME**	**Con VS SP**	**Con TIME VS SP**
**Motile**	42.12 ± 2.42	43.12 ± 1.96	41.19 ± 2.01	n.s.	n.s.	n.s.
**Progressive**	44.28 ± 2.86	43.36 ± 2.80	44.64 ± 2.35	n.s.	n.s.	n.s.
**Rapid progressive**	76.49 ± 0.57	67.26 ± 9.01	66.13 ± 8.84	n.s.	n.s.	n.s.
**Medium Progressive**	40.77 ± 1.89	41.33 ± 2.47	42.62 ± 1.92	n.s.	n.s.	n.s.
**Non Progressive**	35.14 ± 2.31	34.52 ± 1.33	36.89 ± 1.94	n.s.	n.s.	n.s.

**LIN (%)**	**Control Mean ± S.E.**	**Control Time Mean ± S.E.**	**Seminal Plasma Mean ± S.E.**	**Con VS Con TIME**	**Con VS SP**	**Con TIME VS SP**
**Motile**	18.80 ± 0.94	18.56 ± 0.91	18.13 ± 1.22	n.s.	n.s.	n.s.
**Progressive**	19.89 ± 1.39	18.74 ± 1.32	20.27 ± 1.33	n.s.	n.s.	n.s.
**Rapid progressive**	37.24 ± 1.06	29.82 ± 4.19	31.20 ± 4.32	n.s.	n.s.	n.s.
**Medium Progressive**	17.87 ± 0.84	17.80 ± 1.15	19.29 ± 1.12	n.s.	n.s.	n.s.
**Non Progressive**	13.87 ± 0.88	14.08 ± 0.90	15.66 ± 1.14	n.s.	n.s.	n.s.

**WOB (%)**	**Control Mean ± S.E.**	**Control Time Mean ± S.E.**	**Seminal Plasma Mean ± S.E.**	**Con VS Con TIME**	**Con VS SP**	**Con TIME VS SP**
**Motile**	43.88 ± 0.64	42.95 ± 0.81	43.61 ± 0.95	n.s.	n.s.	n.s.
**Progressive**	44.63 ± 0.56	43.11 ± 0.79	44.61 ± 0.81	n.s.	n.s.	n.s.
**Rapid progressive**	48.61 ± 1.32	38.71 ± 5.38	41.23 ± 5.70	n.s.	n.s.	n.s.
**Medium Progressive**	44.13 ± 0.62	43.03 ± 0.77	44.54 ± 0.78	n.s.	n.s.	n.s.
**Non Progressive**	40.28 ± 1.55	41.13 ± 1.42	42.96 ± 1.38	n.s.	n.s.	n.s.

**ALH (μm)**	**Control Mean ± S.E.**	**Control Time Mean ± S.E.**	**Seminal Plasma Mean ± S.E.**	**Con VS Con TIME**	**Con VS SP**	**Con TIME VS SP**
**Motile**	3.26 ± 0.30	3.12 ± 0.30	2.33 ± 0.21	n.s.	**0.0114**	**0.0034**
**Progressive**	3.52 ± 0.27	3.42 ± 0.23	2.98 ± 0.17	n.s.	n.s.	**0.0302**
**Rapid progressive**	3.28 ± 0.19	2.89 ± 0.42	2.50 ± 0.36	n.s.	n.s.	**<0.0001**
**Medium Progressive**	3.54 ± 0.28	3.44 ± 0.24	2.99 ± 0.17	n.s.	n.s.	n.s.
**Non Progressive**	1.45 ± 0.03	1.45 ± 0.04	1.44 ± 0.03	n.s.	n.s.	n.s.

**BCF (Hz)**	**Control Mean ± S.E.**	**Control Time Mean ± S.E.**	**Seminal Plasma Mean ± S.E.**	**Con VS Con TIME**	**Con VS SP**	**Con TIME VS SP**
**Motile**	11.00 ± 0.42	11.25 ± 0.53	9.34 ± 0.72	n.s.	n.s.	n.s.
**Progressive**	11.98 ± 0.43	12.61 ± 0.38	11.93 ± 0.40	n.s.	n.s.	n.s.
**Rapid progressive**	12.78 ± 0.24	10.99 ± 1.51	10.79 ± 1.53	n.s.	n.s.	n.s.
**Medium Progressive**	11.92 ± 0.43	12.68 ± 0.40	11.93 ± 0.40	n.s.	n.s.	n.s.
**Non Progressive**	5.76 ± 0.33	5.80 ± 0.44	6.36 ± 0.42	n.s.	n.s.	n.s.
